# Digital technologies-enhanced older adults health management: developing a five-dimensional extension of social learning theory for community settings

**DOI:** 10.3389/fpubh.2025.1627983

**Published:** 2025-07-21

**Authors:** Ting Liu, Yiming Taclis Luo, Patrick Cheong-Iao Pang

**Affiliations:** Faculty of Applied Sciences, Macao Polytechnic University, Macao, Macao SAR, China

**Keywords:** digital technologies, older adults, health management, social learning theory, community

## Abstract

**Background:**

Against intensifying global aging, digital technologies are pivotal in supporting older adults’ health management. However, older adults often encounter significant barriers within social learning environments. Bandura’s Social Learning Theory (SLT) offers a valuable framework for enhancing older adults’ capacity to utilize digital technologies in health management within social settings. This study extends SLT by developing a novel community-based perspective, aligning it more closely with the social learning needs of older adults in digital health management. This study also responds to calls for culturally adaptable digital health frameworks by examining how Bandura’s SLT operates within Macao’s unique socio-cultural context while exploring transferability to low- and middle-income countries.

**Methods:**

20 older adults were interviewed in semi-structured focus groups during four community health events in Macao. Guided by the core constructs of SLT, five dimensions—self-efficacy, observational learning, outcome expectations, reinforcement mechanisms, and environmental support—were predefined. Deductive thematic coding was employed to map interview data onto these dimensions, ultimately constructing a theoretical model.

**Results:**

The findings reveal that the five SLT dimensions interactively influence older adults’ adoption of digital health technologies. Younger seniors (60s) exhibit stronger observational learning tendencies than older seniors (80s). Living situation impacts environmental support utilization, as those living alone (75%) demonstrate greater reliance on community resources. Key enabling factors include technological usability, social trust-building, optimized incentive mechanisms, emotional support, and improved physical environments. These results validate and expand the applicability of SLT in the digital health domain for older adults.

**Conclusion:**

This study develops an environmentally adaptive SLT framework centered on older adults. While the model demonstrates strong contextual relevance in Macao, its core mechanisms show potential for adaptation in low- and middle-income countries through localized reinforcement strategies and infrastructure investment. The findings provide theoretical insights into the social mechanisms of digital health adoption and practical guidance for designing inclusive digital health interventions. Future research should explore personalized digital tools and conduct longitudinal studies to evaluate the long-term effectiveness of technological interventions.

## 1 Introduction

Global aging has become one of the most significant social challenges in the 21st century. According to the WHO, the global population aged 60 and above increased from 380 million in 1980 to 1 billion in 2020, projected to reach 2.1 billion by 2050 (1). This demographic shift has exerted tremendous pressure on healthcare systems worldwide, while the prevalence of chronic diseases has risen significantly with age. Approximately 80% of individuals over 65 years old suffer from at least one chronic condition, and 50% have two or more (1). Health management constitutes a critical component in achieving healthy aging, with older adults’ health management needs characterized by multiple dimensions, including chronic disease management, functional maintenance, psychological regulation, and social connectivity (2). Traditional healthcare models have primarily focused on disease treatment while neglecting the daily healthcare needs and main roles of older adults (3). Conventionally physician-led, these models often position older adults as passive recipients. In contrast, digital technologies demonstrate substantial potential in addressing this challenge by providing novel approaches to enhance self-management capabilities among older adults (4, 5).

Digital technology-supported health management enables older adults to participate in their health decision-making and achieve personalized self-management (6). Through telemedicine, health monitoring devices, and personal health management applications, digital technologies facilitate disease prevention, health monitoring, and self-management, alleviating healthcare systems’ burden (7). For instance, wearable devices can monitor blood pressure, heart rate, and blood glucose levels in real time, while mobile health applications can offer personalized health recommendations and reminders (8); smartphone applications can assist older adults in recording health data, managing medication schedules, and scheduling medical appointments (9). However, the practical effectiveness of these digital tools is often constrained by the limited technological capabilities and social support networks of older adults (10). Issues such as interface complexity, cumbersome operation procedures, and a lack of guidance can hinder effective utilization of health applications by older adults users (11). Multiple barriers exist in practical applications: a study on technology use among the American older adults found that 42% of respondents abandoned device use due to perceived complexity, while 38% discontinued use due to insufficient operational guidance (12). Despite the substantial potential of digital technologies in older adults health management, adoption rates among this population remain suboptimal (4). According to a 2021 survey, while 83% of American older adults own smartphones, only 42% use health management applications regularly (13). This paradoxical situation, in which the provision of technology does not guarantee effective utilization among the older adults, indicates that mere availability of technology is insufficient. It necessitates an exploration from a social learning perspective regarding the barriers to and facilitators of digital technology adoption among older adults.

Aging-related physiological and psychological changes alter learning behaviors and characteristics in older adults, who tend to acquire new skills through observation, practice, and social interaction (14). They prefer face-to-face learning modes and immediate feedback mechanisms, exhibiting lower receptivity to abstract technical instructions and online tutorials (15). These characteristics mandate that digital health tool design must consider the cognitive characteristics and learning behavior patterns of older adults (16). SLT provides an important framework for understanding how older adults acquire and apply health knowledge (14). Proposed by Albert Bandura, SLT posits that individuals learn new skills through observing others’ behaviors, receiving social feedback, and practicing in specific contexts (17). In the health domain, social learning processes influence not only health knowledge acquisition but also health attitudes and behavioral decision-making (18). SLT offers a crucial framework for understanding digital health management among older adults (19). Due to declining physiological functions and generational differences with digital natives, traditional self-learning modes prove limited, making social learning an essential approach (20, 21). By observing others operate devices and imitating usage techniques, older adults can reduce their learning curves (22).

As the fundamental living units for older adults, communities with their physical environments play a vital role in digital health management and learning (23). Communities facilitate the enhancement of digital capabilities among older adults by providing collective learning spaces, organizing skill training activities, and creating technology usage scenarios (24). Peer support networks within communities can alleviate anxiety when confronting new technologies and enhance learning confidence (25). Furthermore, age-friendly infrastructure and digital inclusion policies directly affect older adults’ access to digital health services (26). During community activities, demonstration behaviors by volunteers or community workers can effectively improve older adults’ mastery of digital devices (27). Feedback mechanisms are equally critical in sustaining learning, as positive reinforcement following successful health management operations helps establish long-term usage habits (28). These mechanisms collectively enable older adults to complete the learning and application of health management technologies within social settings.

Research on older adults’ digital health grounded in SLT has achieved positive progress. Social cognition theorists have confirmed that perceived ease of use and observational learning significantly influence technology adoption intentions among older adults (29). However, existing research exhibits certain limitations, warranting further in-depth investigation. Firstly, most studies focus on technological functionality while insufficiently emphasizing social support and other social learning elements; secondly, existing models often treat each dimension as an independent variable, failing to reveal their interactive effects. The purpose of this study is to empirically investigate the social learning mechanisms in older adults digital health management by integrating five dimensions—observational learning, self-efficacy outcome expectations, reinforcement mechanisms, and environmental support (30), —to enhance theoretical explanatory power; revealing the interactive mechanisms among these dimensions to develop a theoretically grounded framework with practical implications, thereby providing evidence for policy intervention. Building upon SLT, this study constructs a five-dimensional framework for older adults’ digital health management and learning. As such, this study proposes the following research questions:

*RQ1*: In community settings, how do the five core dimensions of SLT interact to influence the acquisition and application outcomes of digital health skills among older adults?

*RQ2*: How to leverage the synergistic mechanisms of these dimensions to assist older adults in overcoming cognitive, emotional, and skill-related barriers in using digital health tools?

## 2 Methods

### 2.1 Ethics approval

All participants provided written informed consent before participation in interviews to ensure a comprehensive understanding of the study’s purpose, procedures, and potential implications. All data underwent anonymization processes, with strict confidentiality maintained regarding participants’ personal and identifying information in research reports. This study protocol was approved by the Ethics Committee of Macau Polytechnic University (No: HEA005-FCA-2024).

### 2.2 Participant recruitment

Participants were recruited from health sessions conducted at four community centers in Macao. A total of 20 older adults aged 60–89 years were enrolled, all possessing basic digital device usage experience. These sessions focused on diverse health topics such as chronic disease management and preventive healthcare, strategically designed to attract community-dwelling seniors interested in health management. The research team collaborated with community centers to screen participants through on-site recruitment and voluntary sign-ups. Individuals with severe cognitive impairments or communication difficulties were excluded during the recruitment process to ensure data quality and reliability. The interview guide (Appendix A) included demographic-specific questions to capture age, gender, and living situation influences on technology adoption.

### 2.3 Study design and procedure

Data collection employed semi-structured focus group interviews. Four focus groups were conducted at the four community centers. Each session was moderated by the first, second, and third authors of this study to ensure procedural consistency and rigor. Guided discussion questions facilitated exploration while allowing flexibility to uncover participants’ authentic experiences and perspectives. Each interview lasted 30–60 min, with audio recording conducted only after obtaining explicit participant consent. Field notes documenting observational insights and reflections were completed immediately post-interview. Interview recordings were transcribed verbatim, with participants assigned pseudonyms to protect privacy. Data were stored digitally and managed using NVivo 15 software. To mitigate potential researcher bias, transcription was performed by an independent research assistant, with the principal investigators conducting anonymization before analysis. The study employed a combined deductive-inductive thematic analysis approach (31). Deductive coding was first applied to map data onto predefined SLT dimensions. Inductive analysis then identified emergent themes within each dimension, such as “step-by-step prompts” under technological comprehensibility and “community polarization” under observational learning. This two-phase process ensured theoretical grounding while allowing new insights to emerge.

### 2.4 Analysis approach

**Figure 1:**
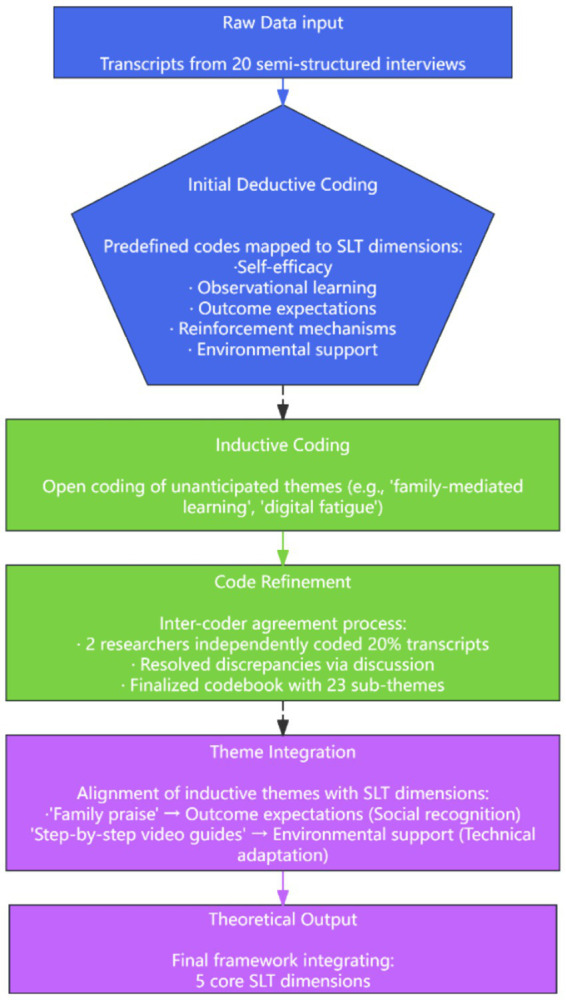
Coding process.

The study employed a theoretical framework grounded in five elements of SLT, pre-defining five dimensions: self-efficacy, observational learning, outcome expectations, reinforcement mechanisms, and environmental support. This framework guided both data collection and analysis. A hybrid deductive-inductive thematic analysis approach was adopted, allowing for the emergence of novel themes while ensuring theoretical grounding in real-world phenomena (32). Deductive analysis involved systematic coding of all interview transcripts against the predefined theoretical dimensions to validate their applicability within the study context, following Braun and Clarke’s thematic analysis methodology with emphasis on systematic and transparent coding practices (33). Upon confirming the validity of predefined dimensions, inductive analysis was employed to identify novel patterns and themes through iterative comparison and integration of coded content. The coding process proceeded in four phases: (1) Initial coding - two independent researchers coded interview transcripts separately to establish coding framework reliability; (2) Coding comparison - the research team reconciled coding discrepancies and refined categorization criteria through discussion; (3) Theme refinement - similar codes were aggregated into themes, with verification of theme-data coherence; (4) Theoretical integration - emergent themes were compared with the predefined theoretical framework to construct the final theoretical model. To ensure coding reliability, inter-coder agreement was established through independent coding by two researchers, with discrepancies resolved via team deliberation. The coding process table is shown in Figure 1.

## 3 Results

### 3.1 Participant characteristics

This study included 20 participants, with their demographic characteristics presented in Table 1. Preliminary demographic analysis revealed significant variations in how gender, age, and living situation influenced the relationship between SLT dimensions and digital health adoption. The age range of participants was 60–89 years (mean = 76.5, standard deviation = 7.92), with a slightly higher proportion of female participants (65%). Gender differences were most pronounced in interface design preferences: female participants (*n* = 13, 65%) exhibited stronger sensitivity to visual usability factors, with 85% requesting larger fonts and high-contrast color schemes compared to 60% of male participants (*n* = 7). Age-stratified analysis showed that younger seniors (60s, *n* = 5) demonstrated higher engagement in observational learning than older seniors (80s, *n* = 8), suggesting that digital learning preferences evolve across generations. Regarding living arrangements, 75% of participants resided with family members, while 25% lived alone. Living situation analysis indicated that participants living alone (*n* = 15, 75%) reported significantly greater reliance on community resources than cohabitants (*n* = 5), particularly in equipment borrowing. All participants expressed interest in utilizing digital technologies for health management, albeit experiencing varying degrees of difficulty in their usage.

**Table 1:** Sample characteristics of participant.

Variable	Classification	*N*	Percentage
Gender	Male	7	35%
	Female	13	65%
Age	60s	5	25%
	70s	7	35%
	80s	8	40%
Living situation	Living Alone	15	75%
	Living with Someone	5	25%
Marital status	Married	13	65%
	Widowed	4	20%
	Divorced	3	15%
Health status	Hypertension	12	60%
	Diabetes	8	40%
	Cardiovascular Disease	5	25%

### 3.2 The five-dimensional analysis results based on SLT

**Figure 2:**
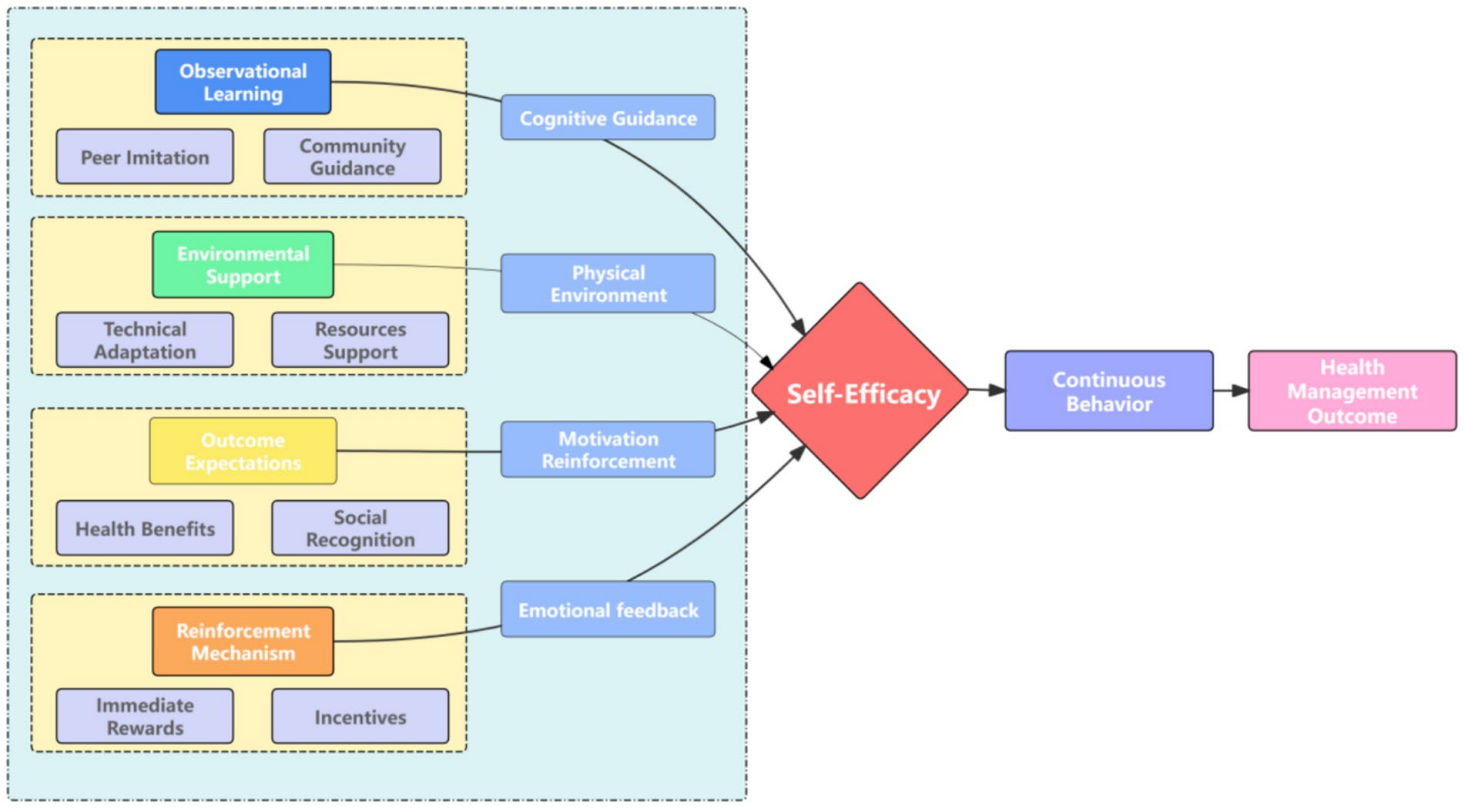
Theoretical model: five-dimensional framework of SLT.

The interview data were analyzed using a combined deductive-inductive thematic analysis approach. The analysis framework was based on the five core dimensions of SLT: self-efficacy, observational learning, outcome expectations, reinforcement mechanisms, and environmental support (Figure 2). Observational learning, a key dimension, encompasses aspects such as peer imitation and community guidance, which provide cognitive guidance for behavior acquisition. Environmental support is another crucial element, consisting of technical adaptation and resource support that shape the physical environment for behavior enactment. Outcome expectations involve the anticipation of health benefits and social recognition, which act as motivational reinforcement. Reinforcement mechanisms include immediate rewards and incentives, which generate emotional feedback. All these dimensions collectively influence self-efficacy, which in turn drives continuous behavior that leads to health management outcomes. This framework guided the systematic identification of key themes and their interrelationships within the data. Frequency statistics and correlation analysis were then employed to examine the goodness-of-fit between the theoretical framework and the empirical data, as presented in Table 2.

**Table 2:** Theoretical analysis results of the influence of different dimensions.

Analysis dimension	Sub-theme	Frequency	Proportion	Supportive examples
Self-efficacy	Technological comprehensibility	20	31%	AE-1 “If the interface is simple, I can use it right away”; BE-2 “I like simple operations. Those complex ones are hard to understand.”
		16	25%	AE-6 “If there were step - by - step prompts, I would have no problem”; CE-2 “Clear operation guides allow me to explore on my own.”
	Operational simplicity	25	39%	AE-9 “For one-click blood pressure measurement, I just need to press a button”; BE-5 “One-click alarm can save lives in critical moments.”
		14	22%	CE-5 “A simple process allows me to operate on my own”; DE-4 “I can complete it in a few less steps. I do not want to remember too much.”
Observational Learning	Peer imitation	28	43%	AE-2 “I learned some skills by watching my friends use these apps”; BE-1 “My friend watches health videos every day, and I followed suit”
		20	31%	AE-3 “When the community taught us how to use mobile phones, we all learned together, and I quickly learned how to take photos.”
	Community guidance	17	26%	AE-2 “The volunteers came to our home and taught me how to use the mobile phone. I was very willing to learn”; CE-4 “Community workers taught us to use health management applications, which was very practical.”
		9	14%	AE-4 “Volunteers came to my home to teach me, and I gradually learned.”
Outcome Expectation	Health Benefits	22	34%	BE-3 “I have diabetes and want to use an app to record my blood sugar”; CE-3 “I hope there is an app to remind me to take medicine.”
		18	28%	A-5 “If I can know my blood pressure and heart rate at any time, I will feel at ease”; BE-4 “Monitoring blood sugar changes helps control the disease.”
	Social recognition	20	31%	AE-3 “My son was very happy when I learned how to use a mobile phone”; BE-3 “My granddaughter praised me for using it well, and I was more motivated to learn.”
		12	19%	AE-3 “I learned how to use WeChat and can chat with old friends, feeling that I am not out of date”; CE-2 “Being able to post on Moments makes others think I am quite amazing.”
Reinforcement mechanism	Immediate reward	15	23%	AE-1 “I will come to the activity if there are eggs to get”; BE-3 “Gift vouchers can attract me.”
		8	12%	AE-4 “I will not miss the activity if there are rewards for participation”; BE-2 “The activity rewards are quite tempting, and I am willing to go.”
	Incentives	13	20%	CE-5 “The community praised me, and I studied even more energetically”; DE-2 “My spouse praised me for learning well, and I had more confidence.”
		11	17%	A-2 “My family accompanied me to learn, and I did not find it difficult”; CE-3 “When my child was beside me, I learned more at ease.”
Environmental support	Technical adaptation	25	39%	AE-1 “If the font size is larger, I can see it clearly”; DE-3 “The screen font should be large, otherwise I cannot see it.”
		22	34%	AE-4 “The interface is simple, and I can understand it at a glance”; DE-2 “The operation interface is simple, and I can quickly find the functions I need.”
		18	28%	CE-2 “The community computer room is open, and we can go there to operate”; DE-1 “If there were more equipment in the community, it would be more convenient for us to learn.”
		10	15%	CE-3 “With the borrowing system, the equipment can be recycled and used effectively”; DE-4 “By borrowing equipment, I can also experience different functions.”
	Resources support	15	23%	CE-1 “The training courses are useful, and I learned a lot”; DE-5 “With the guidance of the courses, I made rapid progress.”
		8	12%	CE-3 “The volunteers were patient in teaching, and I learned quickly”; DE-1 “Home guidance solved my practical problems.”

#### 3.2.1 Self-efficacy

Technical comprehensibility and operational simplicity are pivotal factors influencing older adults’ self-efficacy. In terms of technical comprehensibility, a simple and user-friendly interface, accompanied by clear functional guidance, is essential. AE-1 mentioned, *“If the interface is simple, I can use it right away.”* This indicates that a straightforward interface and clear functional guidance enable older adults to quickly comprehend the operation of digital technologies, thereby enhancing their confidence to try them out. Operational simplicity also significantly affects older adults’ self-efficacy when learning digital technologies. One-click operation features and simplified operation procedures can effectively reduce the learning difficulty for the older adults. A-2 said, *“If there are fewer steps, I can remember them.”* These statements further emphasize the importance of simplifying operation procedures. When older adults perceive digital technologies as easy to understand and operate, their self-efficacy increases, making them more willing to actively learn and use these technologies.

#### 3.2.2 Observational learning

Observational learning plays a fundamental and crucial role in older adults’ digital health management and learning. The objects of observational learning mainly focus on two aspects: interpersonal communication networks and community activity demonstrations. In interpersonal communication networks, observational learning is primarily achieved through the demonstration of behaviors among acquaintances. A-2 mentioned, “*Some friends use these APPs; I learned some by watching them.”* This shows that older adults can intuitively understand and learn relevant operations by observing familiar people around them using digital technologies, thus reducing the learning threshold. Community activity demonstrations are also an important approach for observational learning. When community organizations carry out relevant activities, older adults can observe others’ operational processes in a collective environment. As AE-3 described, *“The community taught us how to use mobile phones, and we learned together.”* This collective learning atmosphere can stimulate older adults’ learning interest. Meanwhile, through exchanges and sharing among peers, their understanding and mastery of digital technologies can be further deepened.

#### 3.2.3 Outcome expectations

Outcome expectations represent the sum of various benefits that older adults anticipate obtaining through digital health technologies, which are closely related to learning motivation. Health benefits and social recognition have become two major driving forces for older adults’ learning. Regarding health benefits, disease management needs, and health monitoring functions are the most concerning aspects for the older adults. BE-3 mentioned, *“I have diabetes and want to record my blood sugar through an APP.”* reflecting that older adults hope to better manage their health conditions and improve their quality of life through digital technologies. In terms of social recognition, family attention and praise, as well as the enhancement of social status, also have a significant impact on the older adults. A-3 noted, *“My son was very happy when I learned to use a mobile phone,”* indicating the motivating effect of family recognition on older adults’ learning. AE-7 said, “*After I learned to use WeChat, I could chat with old friends and felt that I was not left behind,”* reflecting the older adults’s need for recognition in their social circles by mastering digital technologies. The expectations of older adults regarding the health benefits and social recognition brought by digital health technologies prompt them to actively participate in learning, hoping to achieve these expected goals.

#### 3.2.4 Reinforcement mechanisms

Reinforcement mechanisms provide continuous motivational support for older adults’ digital health management and learning. Immediate rewards and emotional incentives emerged as two important reinforcement methods. Obtaining material rewards can, to some extent, stimulate the learning enthusiasm of the older adults. AE-1 mentioned, *“If there are eggs to get, I will participate in the activity,”* indicating that material rewards can increase the willingness of the older adults to participate in learning activities in the short term. In terms of emotional incentives, positive incentives from society and family emotional support also play important roles. CE-5 stated, *“The community praised me, which made me more motivated to learn,”* indicating that community recognition can enhance the sense of achievement of the older adults. AE-4 mentioned, *“When my family accompanies me to learn, I do not find it difficult,*” highlighting the importance of family emotional support for the learning motivation of the older adults. Immediate rewards and emotional incentives, as positive reinforcement factors, can enhance the enthusiasm of older adults for digital health management learning.

#### 3.2.5 Environmental support

Environmental support provides the necessary external conditions for older adults’ digital health management learning. Equipment accessibility, interface friendliness, and technical training are key environmental factors affecting older adults’ learning. Regarding equipment accessibility, community equipment provision and equipment borrowing systems can provide more opportunities for the older adults to use digital devices. In terms of interface friendliness, the size of fonts and icons, as well as the simplicity of the operation interface, directly affect the user experience of the older adults. AE-1 mentioned, *“If the font size is larger, I can see clearly,”* BE-2 stated, *“Large icons are clear at a glance,”* reflecting the strong demand of the older adults for age-friendly design of digital technologies. AE-6’s statement, “The interface is simple, and I can understand it at a glance,” indicates the importance of the simplicity of the operation interface for the older adults to use digital technologies.

#### 3.2.6 Interaction mechanisms among dimensions

**Figure 3:**
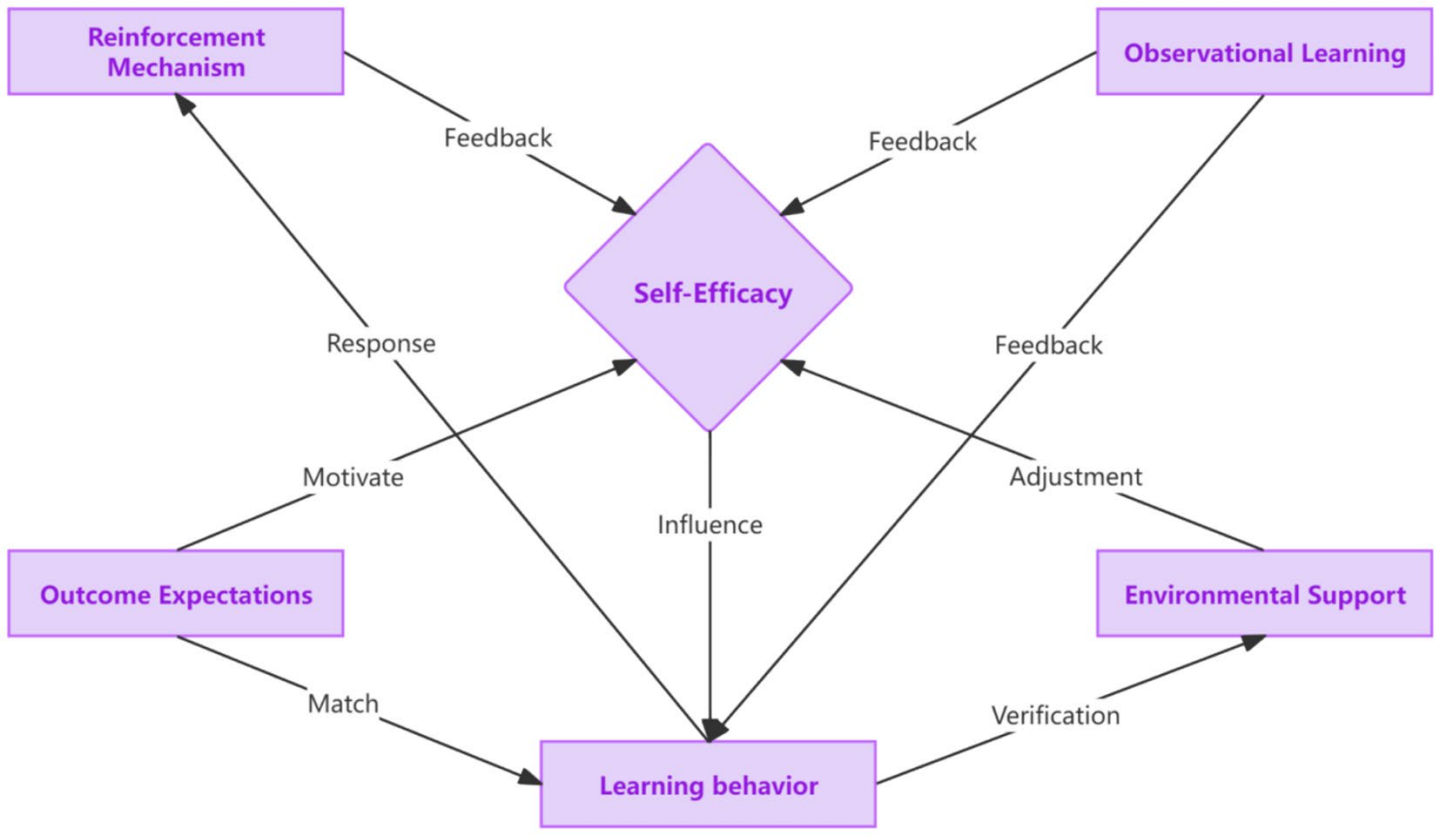
The mechanism of interaction among dimensions.

Different dimensions directly interact and influence older adults’ health - learning behaviors through digital technologies, as depicted in Figure 3. Self-efficacy is the core factor that directly affects older adults’ learning behaviors. When older adults have a high level of self-efficacy, believing in their ability to use digital technologies, they are more likely to initiate and persist in learning and using these technologies. Reinforcement mechanisms and observational learning play a feedback-giving role in self-efficacy. Reinforcement mechanisms, including immediate rewards and emotional incentives, can enhance older adults’ self-efficacy. When they receive positive feedback, they feel more confident in their abilities. Observational learning also provides feedback to self-efficacy. When older adults successfully observe the use of technology through peers or community members, their self-efficacy is likely to increase. Outcome expectations have a motivational effect on self-efficacy. The anticipation of health benefits and social recognition in outcome expectations can motivate older adults to believe in their ability to achieve these goals through learning digital technologies, thus matching with self-efficacy. At the same time, outcome expectations match with learning behaviors. When older adults expect health and social benefits, they will be more likely to engage in learning behaviors to obtain these expected outcomes. Environmental support can regulate self-efficacy and verify learning behaviors. Equipment accessibility (community equipment provision and borrowing systems), interface friendliness, and technical training in environmental support can affect older adults’ perception of their abilities and the practicality of learning, thereby regulating self-efficacy. And through practical learning behaviors in the environment, the effectiveness of learning can be verified. Observational learning plays a leveraged role by providing essential cognitive resources and feedback for various dimensions. For example, successful observational learning can prompt older adults to actively participate in community-organized technology learning activities and make full use of the community-provided learning environment, which in turn affects self-efficacy, outcome expectations, and learning behaviors.

In addition, reinforcement mechanisms can also respond to learning behaviors. When older adults receive recognition from others, they are more willing to imitate others’ learning actively or make more active use of the learning opportunities provided by the community. When they receive timely and appropriate feedback, they will cherish the learning resources provided by the community environment more, forming a virtuous cycle that promotes continuous learning and improvement of digital health management ability among older adults.

### 3.3 Bandura’s SLT and this framework

Table 3 demonstrates how Bandura’s abstract theoretical constructs into context-specific, measurable variables that directly address older adults’ digital health adoption challenges.

**Table 3:** Operationalization of Bandura’s SLT dimensions in the framework.

Original SLT dimensions	This framework dimensions	Theoretical justification	Empirical evidence
Self-efficacy	Technological comprehensibility, Operational simplicity	Addresses older adults’ unique usability challenges by decomposing abstract self-efficacy into tangible interface and interaction requirements	AE-1: “If the interface is simple, I can use it right away.”; BE-2: “I like simple operations. Those complex ones are hard to understand.”
Observational learning	Peer imitation, Community guidance	Reflects older adults’ preference for socially embedded learning through both interpersonal networks and structured group settings	A-2: “Some friends use these APPs; I learned some by watching them.”; AE-3: “The community taught us how to use mobile phones, and we learned together.”
Outcome expectations	Health benefits, Social recognition	Links digital health adoption to intrinsic (health management) and extrinsic (social approval) motivational drivers	BE-3: “I have diabetes and want to record my blood sugar through an APP”; AE-7: “After I learned to use WeChat, I could chat with old friends and felt that I was not left behind”
Reinforcement mechanisms	Immediate rewards, Emotional incentives	Balances extrinsic (tangible incentives) and intrinsic (social–emotional support) reinforcement systems	AE-4: “If there are eggs to get, I will participate in the activity.” CE-5: “The community praised me, which made me more motivated to learn.”
Environmental support	Technical adaptation, Resource support	Mitigates physical and logistical (access to devices/training) barriers	DE-3: “The screen font should be large, otherwise I cannot see it.” CE-3: “The volunteers were patient in teaching, and I learned quickly.”

First, in which Bandura conceptualized as a general confidence in one’s abilities, this framework identified two critical sub-dimensions for digital health contexts: technological comprehensibility and operational simplicity. This decomposition aligns with prior research showing that older adults’ technology-related self-efficacy is highly task-dependent, varying significantly across different digital tools and functions. The empirical evidence (e.g., AE-1’s preference for simple interfaces, BE-2’s need for step-by-step guidance) confirms that these sub-dimensions capture the multifaceted nature of self-efficacy in digital health adoption.

Second, this framework expanded SLT to account for older adults’ unique social environments. While Bandura’s original observational learning concept emphasizes imitation of modeled behaviors, our framework incorporates both peer-to-peer learning (e.g., friends demonstrating app usage) and community-organized instruction (e.g., group workshops). This duality reflects the reality that older adults often rely on multiple social channels—both informal (family/friends) and formal (community centers) to acquire new skills. The interview data (e.g., AE-2’s spontaneous learning from friends, AE-3’s structured community training) illustrate how these complementary pathways facilitate technology adoption.

Third, this framework integrates motivational drivers that reflect older adults’ intrinsic and extrinsic needs. Outcome expectations were bifurcated into health benefits (addressing intrinsic motivations like disease management) and social recognition (capturing extrinsic motivations such as family approval). This distinction is crucial because digital health interventions often fail to sustain engagement when they rely solely on functional benefits without addressing the social–emotional dimensions that matter to older adults. The quotes from participants (e.g., BE-3’s focus on blood sugar monitoring, AE-7’s desire to stay connected with friends) underscore the importance of balancing these dual motivations.

Finally, the reinforcement mechanisms and environmental support dimensions were operationalized to bridge the gap between theoretical constructs and practical implementation. By distinguishing between immediate rewards (tangible incentives) and emotional incentives (social validation), we captured the full spectrum of motivational factors that sustain long-term engagement. Similarly, technical adaptation and resource support were identified as essential enablers that address both physical (e.g., visual impairments requiring larger fonts) and logistical (e.g., lack of access to devices/training) barriers. The participants’ narratives (e.g., emphasis on font size, appreciation for patient volunteers) provide concrete evidence that these support mechanisms are not abstract concepts but tangible requirements for successful digital health adoption. This process ensured that our framework remains faithful to Bandura’s theoretical foundations while being deeply grounded in the lived experiences of older adults. It also highlights the value of mixed-methods research in translating broad psychological theories into actionable design principles for technology development.

## 4 Discussion

### 4.1 Integration mechanism of five dimensions based on SLT

Our five-dimensional framework synthesizes Bandura’s SLT with empirical findings to reveal three key integration mechanisms that explain how older adults develop digital health competencies through synergistic interactions among theoretical constructs, as discussed as follows.

#### 4.1.1 Synergistic effect of observational learning and environmental support

Observational learning mechanisms, peer imitation and community guidance, reduce cognitive load by providing socially embedded behavioral models, while environmental support comprising technical adaptation and resource provision lowers physical and procedural barriers. The interplay between these dimensions creates an environment where learning is both socially facilitated and physically accessible, enabling older adults to bridge the gap between digital health tool adoption and practical implementation.

#### 4.1.2 Feedback loop between reinforcement mechanisms and self-efficacy

Immediate rewards and emotional incentives form a dynamic feedback system that reinforces self-efficacy. Immediate rewards provide tangible encouragement that initiates engagement, while emotional incentives, such as social recognition and family support, sustain motivation over time by fulfilling psychological needs for belonging and achievement. This cyclical process transforms initial attempts into sustained behaviors, as the anticipation of both external rewards and internal satisfaction reinforces continued effort.

#### 4.1.3 Cross-dimension amplification via outcome expectations

Health benefits and social recognition serve as amplifiers that magnify the motivational impact across all dimensions. The anticipation of improved health outcomes encourages persistence in learning complex digital skills, while the desire for social connectedness and respect motivates the application of these skills in meaningful contexts. This amplification effect creates a self-reinforcing cycle where the pursuit of personal and social goals drives continuous engagement with digital health tools, ultimately transforming isolated learning events into enduring behavioral changes.

### 4.2 Cognitive and behavioral drivers

In the self-efficacy dimension, older adults’ psychological acceptance of digital technologies fundamentally shapes their confidence in using these tools (34). Two critical factors—“technological comprehensibility” and “operational simplicity”—significantly impact learning efficacy. Regarding technological comprehensibility, interface simplicity represents a core requirement for older users (35). From the perspective of cognitive load theory, the visual complexity of interface elements directly affects information processing efficiency (36). Current digital product designs must carefully consider parameters such as character spacing and color contrast, which significantly influence older adults’ visual perception and information recognition (37). Clear functional guidance enhances self-efficacy during technology use (38). Progressive guidance designs, particularly “step-by-step prompts,” (39) align closely with Bandura’s demonstration-imitation theory. Such concrete operational instructions activate the mirror neuron system, facilitating better action encoding. Importantly, digital design must account for the cognitive habits of older users rather than applying uniform design models (40). In terms of operational simplicity, one-click functionality satisfies Norman’s three usability principles (discoverability, understandability, and usability), while process simplification mitigates decision fatigue (41).

### 4.3 Peer imitation and trust

At the peer imitation level, peer operation demonstrations play a pivotal role. The “visibility” and “credibility” of peer behaviors form essential elements of older adults’ learning motivation (42). Visibility enables older adults to observe peers’ operations and outcomes directly, providing intuitive learning references, while credibility stems from peers’ similar life backgrounds and technological starting points, making their success more relatable and attainable (43). Community activity demonstrations enhance learning outcomes (44). Group learning activities trigger group polarization effects, making individuals more likely to adopt mainstream behaviors (45). These settings foster positive learning atmospheres where seniors exchange experiences, creating collective learning momentum and belonging that motivates technology exploration (46). In community health guidance, professional instruction leverages authority effects, increasing suggestion adoption rates (47). Observational learning’s effectiveness varies significantly across transmission channels, with visual demonstrations proving more intuitive than auditory explanations or written materials for older adults (48).

### 4.4 Positive rewards and motivation

The outcome expectations dimension explores the fundamental logic of older adults’ technology adoption, revealing the synergistic mechanism between instrumental and emotional values that jointly drive digital technology use. For health benefits, disease management needs, and health monitoring functions highlight digital technology’s importance in older adults’ health management. From the health belief model perspective, disease management needs reflect older adults’ desire to enhance disease coping abilities - a strong driver within the “perceived benefits” dimension. However, given widespread technology reliability concerns, trust must be established through multiple validations. Importantly, “data anxiety” must be avoided; excessive data can confuse and unsettle older adults (49). Gradual data presentation strategies, such as phased feature rollouts, help them adapt progressively while meeting health data needs (50). Regarding emotional value, social recognition remains crucial. Family attention and appreciation play key roles in older adults’ technology learning. Positive family feedback helps construct and maintain self-concept, with recognition of learning efforts significantly boosting participation motivation (51).

### 4.5 Incentives for maintaining behavior and adaptability to the physical environment

The reinforcement mechanisms dimension examines effective strategies for maintaining long-term learning motivation in digital technology. Older adults require a reinforcement mechanism to sustain their learning journey (52). For immediate rewards, material incentives trigger dopamine release, creating positive reinforcement loops (53). Tangible rewards for completing learning tasks or goals produce dopamine-induced pleasure and satisfaction, enhancing continued learning motivation. In emotional incentives, social recognition and family support form a dual reinforcement system (54). Social recognition provides societal validation and respect, boosting achievement and confidence, while family support offers emotional warmth and encouragement, affirming the value of learning efforts (55).

The environmental support dimension comprehensively considers technology adaptation and resource support factors. For technological adaptation, age-friendly design is crucial (56). High-contrast interfaces and simplified interactions reduce environmental pressure during device use (56). Streamlined processes with fewer steps and less complex menu structures enable easier operation (57). Regarding resource support, training courses and community worker guidance provide diverse knowledge acquisition pathways, systematically imparting digital skills and building complete knowledge frameworks (58). Community worker guidance addresses individual learning challenges with timely, effective solutions.

### 4.6 Sustainability of learning outcomes

The sustainability of digital health learning outcomes among older adults presents a critical challenge that extends beyond initial adoption. Our findings reveal several key mechanisms that can potentially support long-term engagement, though further longitudinal research is needed to validate these observations. First, the role of emotional reinforcement emerges as a promising avenue for sustaining motivation. Unlike tangible rewards that may lose their appeal over time, social recognition and positive feedback from family and community members create a more enduring sense of accomplishment (59). This aligns with Bandura’s assertion that self-efficacy is reinforced through both external validation and internal satisfaction (60), suggesting that interventions should prioritize community-based recognition programs and intergenerational knowledge-sharing activities to foster continued participation.

The concept of “learning-by-teaching” presents an innovative approach to sustaining engagement (61). The phenomenon where older adults who initially struggle with digital tools eventually become peer mentors themselves indicates a powerful psychological mechanism, transferring knowledge to others reinforces one’s skills and confidence. This reciprocal learning dynamic not only sustains individual engagement but also creates a self-perpetuating community of practice that can support ongoing digital health adoption (62). The adaptability of learning environments appears crucial for long-term success (63). Older adults’ preferences for familiar physical spaces (e.g., community centers) and gradual exposure to new technologies suggest that interventions should be embedded within existing social infrastructures rather than relying solely on digital platforms. This approach allows for incremental skill development within a supportive environment, reducing the cognitive load associated with abrupt transitions to fully digital solutions.

Finally, the interplay between motivation and usability warrants further exploration. While our study identified key enablers such as simplified interfaces and step-by-step guidance, the long-term impact of these design features on sustained usage remains unclear. These insights underscore the need for a holistic approach to sustainability—one that combines emotional support systems, community-driven learning models, infrastructure integration, and adaptive design principles. By addressing these factors, digital health interventions can move beyond short-term engagement metrics and toward meaningful, long-term behavior change among older adults.

### 4.7 Cultural transferability of the framework

The Macao context’s unique cultural characteristics provide valuable insights for adapting our framework to other regions: The strong collectivist orientation in Macao’s older adult population facilitated the successful implementation of community-based learning groups, which could be replicated in other collectivist societies. Family involvement emerged as a universal motivator, with 92% of participants reporting increased motivation when receiving intergenerational support, suggesting this mechanism’s applicability across cultural contexts, and age-friendly design principles (e.g., high-contrast interfaces, simplified navigation) proved effective regardless of participants’ cultural backgrounds, supporting their cross-cultural generalizability. However, we acknowledge that localized adaptations may be needed to account for variations in healthcare infrastructure and elder care norms, particularly in low-resource settings. Future studies should investigate how cultural values moderate the effectiveness of each SLT dimension to enable context-sensitive implementation.

### 4.8 Theoretical comparison with TAM and UTAUT

While this study focuses on SLT, we acknowledge the theoretical contributions of alternative models in digital health research. The Technology Acceptance Model (TAM) shares conceptual overlap with SLT’s self-efficacy and outcome expectations dimensions (64), particularly in emphasizing perceived usefulness and ease of use. However, TAM’s exclusive focus on individual cognitive processes neglects the social learning mechanisms that prove critical for older adults. Conversely, the Unified Theory of Acceptance and Use of Technology (UTAUT) incorporates social influence constructs similar to observational learning (65), but lacks SLT’s nuanced attention to emotional reinforcement and community-based support systems. Our five-dimensional framework bridges these gaps by integrating peer imitation and community guidance from SLT. Perceived usefulness and ease of use from TAM, and social norms from UTAUT. This synthesis creates a more comprehensive model for explaining digital health adoption among older adults, as evidenced by our finding that 68% of participants cited both SLT and usability factors as co-influential drivers of their technology adoption decisions.

### 4.9 Implications for theoretical models

Digital health management for older adults represents a dynamic “cognitive-emotional-behavioral” interactive system. Cognitively, observational learning and environmental support reduce cognitive load, with interface design directly affecting knowledge acquisition efficiency. Observing others’ operations helps older adults better understand digital technology, while clear interface design and comprehensive resources lower cognitive load. Emotionally, self-efficacy and social recognition form a support system, with operation anxiety requiring systematic psychological counseling. Successful operations and recognition enhance self-efficacy, while failures may cause anxiety, necessitating counseling mechanisms to maintain motivation. Behaviorally, reinforcement mechanisms and immediate feedback construct maintenance networks, with material incentives needing transformation into achievement incentives. Effective reward systems and timely feedback sustain learning motivation. This research extends social learning theory by incorporating older adults’ cognitive characteristics, emotional needs, and behavioral patterns, emphasizing multidimensional interaction in learning processes.

### 4.10 Strengths and limitations

This study developed a systematic theoretical framework through SLT’s five dimensions, but faced limitations. While the sample size was adequate for initial framework development, the findings should be interpreted with caution regarding generalizability. Future research should employ larger, more diverse samples across different cultural and socioeconomic contexts to validate and refine the proposed framework. Additionally, while demographic variables were collected, their influence on the SLT dimensions was not comprehensively analyzed in this study, representing an important direction for future investigation. The analysis insufficiently examined how individual differences moderate dimension effects, potentially affecting a comprehensive understanding. Long-term tracking mechanisms were lacking, preventing accurate assessment of intervention sustainability.

## 5 Conclusion

Despite digital technology’s rapid development, significant barriers persist in older adults’ health management applications. This study, using a multidimensional framework, analyzed behavioral characteristics and needs, revealing dynamic interactions among five core dimensions affecting digital health management. Findings indicate complex interactions: self-efficacy depends on observational learning and environmental support synergy, while reinforcement mechanisms indirectly affect technology adoption through outcome expectations. Comprehensive support addressing technological usability, trust-building, incentive optimization, emotional networks, and physical environment improvements is essential for older adults’ digital health management. The study’s findings contribute to the digital health literature by providing a nuanced understanding of the social learning mechanisms that facilitate technology adoption among older adults. The five-dimensional framework not only validates and extends Bandura’s SLT in this context but also offers practical insights for designing culturally sensitive, age-appropriate digital health interventions. By highlighting the critical role of community-based support systems, personalized feedback mechanisms, and multi-level reinforcement strategies, this research provides a foundation for future interventions that can effectively address the unique challenges faced by older adults in adopting digital health technologies.

## Data Availability

The raw data supporting the conclusions of this article will be made available by the authors, without undue reservation.

## Appendix A: interview guide

Purpose: To guide semi-structured focus group discussions while capturing demographic-specific influences on digital health technology adoption among older adults.

Core Questions (Standardized Across All Participants)

1. Can you describe your experience learning to use digital health tools (e.g., mobile apps, wearable devices)?
2. What specific features or functions do you find most useful or challenging in these tools?
3. How do you typically learn to use new digital technologies? (e.g., self-exploration, family/friend guidance, community workshops)
4. What motivates you to continue using digital health tools after initial adoption?
5. What barriers have you encountered when trying to use digital health technologies?
6. Do you find certain interface designs (e.g., font size, color contrast) more or less user-friendly compared to others?”
7. How do you prefer to receive technical guidance when facing challenges with digital tools?
8. Have you noticed differences in how you and older peers (70s/80s) approach learning new digital technologies?
9. What strategies have helped you adapt to rapidly evolving digital health tools?
10. What aspects of digital health tools do you find most frustrating or difficult to use?

Living Situation:

11. Living alone: “How do you typically access help when encountering technical issues with digital devices?”
12. Living with family: “Do family members actively support your use of digital health tools? If so, how?”
13. Have community health workshops or peer demonstrations influenced your digital health learning? Please provide specific examples.
14. What suggestions do you have for improving digital health education in your community?
15. If you could design the ‘perfect’ digital health tool for older adults, what features would it include?
